# Outcomes of percutaneous musculotendinous lengthening in children with Cerebral Palsy: a systematic review

**DOI:** 10.1590/1984-0462/2026/44/2025294

**Published:** 2026-06-22

**Authors:** Ana Paula Tedesco, Renata D’Agostini Nicolini-Panisson

**Affiliations:** a Instituto de Neuro-Ortopedia, Caxias do Sul, RS, Brazil.; b Centro Universitário da Serra Gaúcha, Caxias do Sul, RS, Brazil.

**Keywords:** Cerebral palsy, Minimally invasive surgery, Tenotomy, Orthopedic procedures, Paralisia cerebral, Procedimentos cirúrgicos minimamente invasivos, Tenotomia, Procedimentos ortopédicos

## Abstract

**Objective:**

The aim of this study was to analyze the outcomes — joint range of motion, functional aspects, anatomical aspects, and complications — of percutaneous musculotendinous lengthening in children with cerebral palsy.

**Data source:**

In total, six databases were searched (Medline/PubMed, Cochrane Library, Physiotherapy Evidence Database/PEDro, LILACS, Biblioteca Virtual em Saúde/BVS, and SciELO) for publications from 2004 to 2024 on percutaneous musculotendinous lengthening in children with cerebral palsy (0–18 years), with ≥1 year of follow-up, in English or Portuguese. The quality of nonrandomized studies was assessed using the Newcastle-Ottawa Scale, and that of randomized trials using the Cochrane risk-of-bias tool.

**Data synthesis:**

Of 151 studies identified, 14 met the inclusion criteria, including three randomized trials. Methodological limitations were frequent, including short follow-up periods, demographic heterogeneity, nonuniform evaluation methods, and the lack of confounder control. Only three studies used three-dimensional gait analysis. Notably, seven studies on hamstring percutaneous musculotendinous lengthening showed functional and range-of-motion improvements. Comparisons with open techniques showed no consistent kinematic differences. Evidently, three studies on adductor percutaneous musculotendinous lengthening found increased hip abduction, with no additional benefit from subsequent open procedures. Notably, the literature regarding Achilles issues is limited to three studies; of these, a single long-term clinical trial identified a 43% recurrence rate without calcaneus deformity, whereas the remaining two were restricted to anatomical and compartment pressure-related issues. Significantly, one study confirmed lower costs and blood loss in comparison with open procedures. The use of post-op immobilization and complications — overcorrection, recurrence, nerve and muscle injury, and increased pelvic tilt — were reported.

**Conclusions:**

Percutaneous musculotendinous lengthening may offer benefits, but stronger evidence from high-quality, long-term studies is needed to confirm its effectiveness.

## RESUMO

**Objetivo:**

Analisar os desfechos: amplitude de movimento, aspectos funcionais, anatômicos e complicações cirúrgicas do alongamento musculotendinoso percutâneo em crianças com paralisia cerebral.

**Fontes de dados:**

Seis bases de dados (Medical Literature Analysis and Retrieval System Online – Medline/PubMed, Cochrane Library, Physiotherapy Evidence Database – PEDro, Latin American and Caribbean Health Sciences Literature – LILACS, Biblioteca Virtual em Saúde – BVS e Scientific Electronic Library Online – SciELO) foram pesquisadas, para estudos entre 2004 e 2024, sobre alongamento musculotendinoso percutâneo em crianças com paralisia cerebral (0–18 anos), com seguimento ≥1 ano. A qualidade dos estudos foi avaliada pela *Newcastle-Ottawa Scale* e pela *Cochrane risk-of-bias*.

**Síntese dos dados:**

Quatorze estudos foram incluídos, sendo três ensaios randomizados. Limitações metodológicas foram frequentes: curto tempo de seguimento, variabilidade demográfica, métodos de avaliação não uniformes e ausência de controle de fatores de confusão. Apenas três estudos utilizaram análise tridimensional de marcha. Sete estudos sobre alongamento percutâneo de isquiotibiais mostraram melhora funcional e de amplitude de movimento. Comparações com técnicas abertas não mostraram diferenças cinemáticas consistentes. Três estudos sobre alongamento percutâneo musculotendinoso dos adutores demonstraram aumento da abdução do quadril, sem benefício adicional com procedimentos abertos subsequentes. Três estudos abordaram o tendão de Aquiles: apenas um clínico de longo prazo, com 43% de recorrência sem deformidade do calcâneo; os outros dois trataram de questões anatômicas e pressão compartimental. Um estudo confirmou menor custo e perda sanguínea com a técnica percutânea. Foram relatados aspectos da imobilização pós-operatória e complicações: hipercorreção, recorrência, lesão nervosa e muscular e aumento da inclinação pélvica.

**Conclusões:**

O alongamento musculotendinoso percutâneo pode oferecer benefícios, mas evidências mais robustas e de longo prazo são necessárias para confirmar sua efetividade.

## INTRODUCTION

 Cerebral palsy (CP) is a nonprogressive motor disorder from early brain injury, often linked to prematurity and low birth weight, affecting 2–3 per 1000 live births.^1^ It leads to lifelong motor impairments due to altered neural input and muscle structure. The spastic type accounts for 75% of cases and involves pyramidal tract injury and velocity-dependent stiffness.^1^ Limb involvement varies, and mobility is classified by the Gross Motor Functional Classification System (GMFCS).^2,3^

 Although neurologically based, treatment frequently targets muscle contractures — especially in biarticular muscles — due to structural changes that limit joint range of motion (ROM).^1^ Surgical options include open and percutaneous techniques such as tenotomy, intramuscular lengthening, and myofascial release. Percutaneous musculotendinous lengthening (PML) has gained interest as a minimally invasive option with less pain, faster recovery, and preserved strength compared to open procedures. Early studies suggest benefits in gait, gross motor function, and ROM, though long-term data remain scarce.^4^

 This review examines current literature on PML in children with CP, focusing on methodology and outcomes: ROM, functional aspects (including gait findings), anatomical aspects, and complications. A preliminary search found no existing systematic or scoping reviews on the topic. 

## METHOD

**Figure 1:**
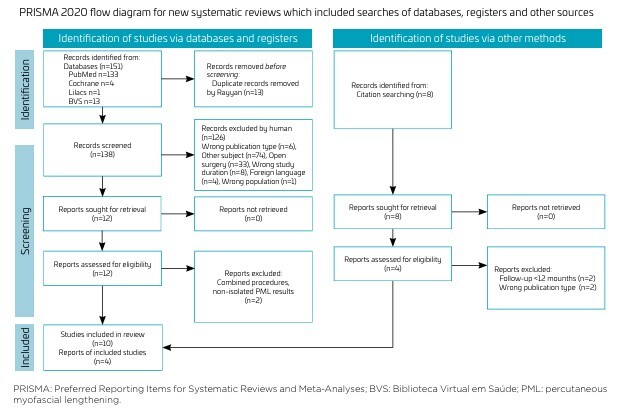
Flow diagram of the study selection process according to PRISMA 2020.^5^

 Preferred Reporting Items for Systematic Reviews and MetaAnalyses (PRISMA) Guidelines 2020 were used for this systematic review.^5^ A flowchart summarizing article selection is presented in Figure 1. An initial search of Medline/PubMed was performed to identify relevant articles. Terms from titles, abstracts, and indexing were used to build a full strategy across six databases: Medline/PubMed, Cochrane Library, PEDro, LILACS, BVS, and SciELO. 

 The MEDLINE strategy combined controlled vocabulary (MeSH terms) and free-text terms: "Cerebral palsy"[MeSH Terms] AND 2004/01/01:2025/12/31[Date – Publication] AND (("minimally invasive surgical procedures"[MeSH Terms] OR "tenotomy*"[MeSH Terms] OR (("percutaneous"[All Fields] OR "percutaneously"[All Fields] OR "percutaneous"[All Fields]) AND "muscle lengthening"[Text Word]) OR "myofascial lengthening"[Text Word] OR "myofascial lengthening"[Text Word] OR (("select"[All Fields] OR "selectability"[All Fields] OR "selectable"[All Fields] OR "selected"[All Fields] OR "selecting"[All Fields] OR "selection s"[All Fields] OR "selection, genetic"[MeSH Terms] OR ("selection"[All Fields] AND "genetic"[All Fields]) OR "genetic selection"[All Fields] OR "selection"[All Fields] OR "selectional"[All Fields] OR "selections"[All Fields] OR "selective"[All Fields] OR "selectively"[All Fields] OR "selectives"[All Fields] OR "selectivities"[All Fields] OR "selectivity"[All Fields] OR "selects"[All Fields]) AND "percutaneous lengthening"[Text Word]) OR "percutaneous surgery"[Text Word] OR "percutaneous tenotomy"[Text Word] OR "percutaneous tenotomy"[Text Word] OR "subcutaneous tenotomy"[Text Word]) AND 2004/01/01:2025/12/31[Date - Publication]). Search terms were adapted for the other databases: PEDro and Cochrane Library were queried using "cerebral palsy percutaneous muscle lengthening"; SciELO using "*paralisia cerebral AND alongamento muscular percutâneo*"; and LILACS/BVS using "cerebral palsy AND percutaneous muscle lengthening". 

 The PICO question was: *What are the outcomes of PML in individuals with CP regarding ROM, functional results and complication rate, specific anatomical findings, and the comparison with other surgical methods of lengthening?* The RAYYAN® program was used for article selection. References from included studies were also screened. Studies in English or Portuguese, published from 2000 to 2024, were included. The primary outcomes were joint ROM; functional results assessed using the Gross Motor Function Measure (GMFM), GMFCS, Functional Independence Measure (FIM), and Functional Mobility Scale (FMS); gait parameters assessed by 3D gait analysis; and surgical complications such as hypercorrection, recurrence, nerve injury, or bleeding. 

 Notably, two independent reviewers (A.P.T. and R.N.P.) screened articles by title and abstract using RAYYAN®. Full texts were read when necessary, and disagreements were resolved by discussion or with a third reviewer. This review included studies reporting on PML in children with CP (0–18 years), evaluating clinical or functional outcomes with at least 1 year of follow-up, or describing surgical anatomy. All study types were eligible, except letters and case reports. Data verification: Study data (type, evidence level, GMFCS, age at surgery, procedure, methods of evaluation, follow-up, outcomes, complications) were extracted independently by two reviewers and cross-checked. Discrepancies were resolved by consensus. Data were confirmed against original articles when needed. Studies involving needle tenotomy, non-peer-reviewed publications, or combined procedures without isolated PML analysis were excluded. 

 Methodological quality was assessed by the same reviewers using the Newcastle-Ottawa Scale (NOS)^6^ for observational studies and Version 2 of the Cochrane risk-of-bias tool for randomized controlled trials (RCTs; RoB 2).^7^ Evidence levels were classified according to the Oxford Centre for Evidence-Based Medicine scale (OCEBM).^8^

 In addition to evaluating methodological quality using NOS and RoB 2, the GRADE approach was considered to assess the overall certainty of the evidence across studies. Given the heterogeneity of outcome measures, limited quantitative data, and variable reporting, GRADE was applied narratively, focusing on key domains including risk of bias, inconsistency, indirectness, imprecision, and publication bias. 

## RESULTS

 In total, 14 studies were included, as shown in the PRISMA Flow Diagram (Figure 1)^5^. Table 1 ^9-22^, details their characteristics and quality. 

**Table 1:** Characteristics, level of evidence, and quality of the included studies.

Study	Goal of study	Type of study	NOS	RoB 2	Level of evidence
Afaque et al.^19^	Comparing open vs PML hamstring in spastic diplegia	Randomized controlled trial		Some concerns	II
Ariyawatkul et al.^9^	To measure neurovascular proximity, using ultrasound during PML hamstrings	Prospective observational	High quality: 8		III
Carbonell^10^	To measure pressure of the posterior superficial compartment of legs before and after a PML Achilles tendon	Prospective observational	Moderate quality: 5		III
El Hage et al.^20^	To evaluate the effectiveness and safety of PML adductor longus compared to open procedure	Randomized controlled trial		Low risk	II
Erdal et al.^11^	To assess neurovascular safety after PML Achilles tendon	Retrospective cohort	Moderate quality: 6		III
Gordon et al.^14^	To assess gait outcomes after PML hamstrings	Retrospective cohort	Moderate quality: 6		IV
Hachache et al.^22^	To evaluate the effectiveness and safety PML adductor longus and proximal gracilis compared to open.	Prospective comparative cohort	High quality: 7		II
Krupinski et al.^15^	To evaluate PML Achilles tendon	Retrospective cohort	Moderate quality: 6		IV
Mansour et al.^21^	To compare PML with open hamstring lengthening	Randomized controlled trial		Low risk	II
Mitsiokapa et al.^16^	To evaluate functional impact of PML (adductors, hamstrings).	Retrospective cohort	High quality: 7		IV
Mozafari et al.^12^	To compare open versus PML hamstring	Retrospective cohort	High quality: 8		III
Nazareth et al.^13^	To assess functional outcomes of PML vs open hamstring lengthening	Retrospective cohort	High quality: 8		III
Pierz et al.^17^	To assess safety and outcomes of PML hamstring	Retrospective cohort	High quality: 7		IV
Wild et al.^18^	To evaluate clinical outcomes after PML hamstring	Prospective case series	Moderate quality: 6		IV

Notes: The quality of the observational studies analyzed with New Castle-Ottawa Quality Assessment Scale. The Version 2 of the Cochrane risk-of-bias tool for randomized trials (RoB 2) was used to assess the randomized controlled trial studies. Level of evidence estimated according to the Oxford Centre for Evidence-Based Medicine Scale (OCEBM).

NOS: Newcastle-Ottawa Scale; PML: Percutaneous Musculotendinous Lengthening.

 The methodological quality of the included studies was assessed using multiple tools (Table 1). According to OCEBM, most studies were classified as level III (n=5)^9-13^ or level IV (n=5),^14-18^ with three RCT rated as level II.^19-21^ Observational studies assessed by NOS were considered high quality (n=6)^9,12,13,16,17,22^ or moderate quality (n=5).^10,11,14,15,18^ The three RCTs evaluated using the Cochrane RoB 2 tool were judged to have low risk of bias (n=2)^20,21^ and some concerns (n=1).^19^ Additionally, a narrative application of the GRADE framework was performed to assess the overall certainty of evidence across studies. Due to marked heterogeneity in outcomes, inconsistent reporting, and limited quantitative data, the overall confidence in the evidence was considered low to very low, particularly due to risk of bias, inconsistency, and indirectness. 

 Participant demographics are in Table 2 ^9-22^. In total, two studies focused only on diplegia,^12,19^ and eight studies included hemiplegia and quadriplegia as well.^9,10,13-17,21^ Studies varied in age and GMFCS distribution. 

**Table 2:** Participant profile and evaluation criteria of the included studies.

Study	n	Age	Type of spastic CP	GMFCS	Follow-up (months)
Afaque et al.^19^	100	9. 5±1.8	Diplegia	I–V	12
Ariyawatkul et al.^9^	16 (32 knees)	7.9	Diplegia, quadriplegia	--	N/A
Carbonell^10^	18 (28 legs)	9.1	Quadriplegia, hemiplegia	III–IV	N/A
El Hage et al.^20^	27 (50 hips)	--	--	--	N/A
Erdal et al.^11^	19 (30 ankles)	11.7	--	--	17.6 (12–26)
Gordon et al.^14^	48	9.5	Diplegia, hemiplegia, quadriplegia	I–III	SFU: 11.8 (8.0–17.4) LFU: 33.8 (18.4–69.5)
Hachache et al.^22^	31 (59 hips)	8.5	--	I–V	N/A
Krupinski et al.^15^	53 (76 feet)	7	Diplegia, hemiplegia	I–II	*10,9 years (3–17)
Mansour et al.^21^	18 (31 knees)	8.5	Diplegia, hemiplegia, paraplegia	--	N/A
Mitsiokapa et al.^16^	53	3-12	Diplegia, quadriplegia, hemiplegia	I–IV	24 (6–24)
Mozafari et al.^12^	54 (108 knees)	8.5	Diplegia	I–V	Open:19.1 (12–49)PML: 18.3 (14–45)
Nazareth et al.^13^	87 (open: 65 PML: 22)	8.3	Diplegia, hemiplegia	I–IV	29.4±19.9 Open: 33.1 PML: 19.2
Pierz et al.^17^	52	8.2	Diplegia, hemiplegia	I–III	10-49
Wild et al.^18^	201 (Phase 1: 17, Phase 2: 184)	7. 6	--	II–IV	Phase 1:6,3(5–13) Phase 2:33,3(27–38)

Notes: Age expressed in mean age at surgery, except Mitsiokapa et al.^16^ expressed in range. For Erdal et al.,^11^ age was reported as a range in months and was converted to years to allow comparison with other studies presenting mean age in years. Follow-up expressed in months, except *, expressed in years. GMFCS is expressed by a range.

CP: cerebral palsy; GMFCS: Gross Motor Function Classification System; N/A: not applicable; SFU: short follow-up; LFU: long follow-up; PML: percutaneous musculotendinous lengthening.

 Evaluation criteria: included magnetic resonance imaging (MRI),^11^ ultrasound,^9^ and clinical measures: hip abduction,^20,22^ ankle dorsiflexion,^15^ popliteal angle (PA),^12,14,19^ ROM,^19^ GMFCS,^12,16^ FIM,^18,19^ Observation Gait Scale (OGS),^19^ Physician Rating Scale (PRS),^19^ gait assessment,^12,15^ FMS,^18^ GMFM,^16^ compartment pressure,^10^ lower limb and foot observation,^18^ parent satisfaction,^15^ and tridimensional (3D) gait analysis.^13,14,17^ The latter included Gait Profile Score (GPS); knee flexion at initial contact (KFIC), knee extension at initial contact (KEIC), maximal knee extension in stance, gait speed, pelvic tilt, and Gait Deviation Index (GDI). 

Table 3 ^9-22^ summarizes surgical details and outcomes, and Table 4 ^9-22^ presents complications. Most studies had mean follow-ups of nearly 2 years; only one reached 10 years.^15^ Concomitant procedures (bone surgery, soft tissue, nerve blocks) were mentioned in four studies.^13,14,17,19^

**Table 3:** Procedures and results of the included studies.

Study	Procedures	Results
Afaque et al.^19^	Open vs PML hamstring	Within-group improvements in GMFCS, FIM, PRS, OGS, PA; no significant difference between groups.
Ariyawatkul et al.^9^	PML hamstring, ultrasound	High risk of peroneal nerve proximity to the lateral hamstring; open technique recommended for the lateral side.
Carbonell^10^	PML Achilles tendon, compartment pressure	Pressure of superficial posterior compartment of leg higher in tetraplegia than hemiplegia; significantly decreased after surgery; correlated with degree of plantar flexion.
El Hage et al.^20^	PML adductor longus followed by open and completion of tenotomy as necessary.	Hip abduction improved after PML; no further gain with open procedure.
Erdal et al.^11^	PML Achilles tendon, postoperative MRI	Sural nerve — enlargement with degenerative changes: 16,7%, complete transection: 3,3%. Lateral cut >3 cm proximal to Achilles insertion: higher risk; medial side: safe (≥5 mm distance from tendon)
Gordon et al.^14^	PML hamstring	Gait analysis: Both groups: improved: KEICd, velocity, GDI; increased stride length and decreased PA. SFU: increased anterior pelvic tilt and knee extension, and decreased plantarflexion at initial contact.
Hachache et al.^22^	PML adductor longus and gracilis followed by open	Improvement of abduction after PML; no further gain after open procedure. Bleeding problems.
Krupinski et al.^15^	PML Achilles tendon	On clinical observation: Recurrence: 43% (4%<8 years old vs 72% >8 years); good cosmetic and satisfaction outcomes; no calcaneal deformity observed.
Mansour et al.^21^	PML of medial hamstrings, followed by open exploration	Gain in PA less after PML, undesirable cut of the semimembranosus muscle more than 50%: 25%, complete rupture: 19%.
Mitsiokapa et al.^16^	PML adductors, hamstrings, fascia lata, sartorius+obturator blocks	GMFCS: improved by 1 level in 34 cases and 2 in 5. GMFM improved 71.19% to 83.19%; all patients except one continued improving GMFM at 24 months.
Mozafari et al.^12^	Open vs PML hamstring+concomitant surgeries	PML associated with shorter surgical time, hospital stay, and lower cost; both techniques improved PA and GMFCS, with no difference between groups.
Nazareth et al.^13^	Open vs PML hamstring+concomitant surgeries	Gait analysis and PA: No difference in ROM or stance phase knee kinematics between groups, increase of anterior pelvic tilt >10°: PML 23%, open 14%.
Pierz et al.^17^	PML hamstring	Gait analysis and clinical exam: Both improved in PA, static knee extension, KEICd, mean knee flexion in stance; GMFCS III: increase in pelvic tilt.
Wild et al.^18^	PML adductors, hamstrings, gastrocnemius	Phase 1 (video analysis): improved knee/ankle ROM during gait and FMS 5, 50, 500. Phase 2 (telephone interview): FMS 500 continued improvement; FMS 5,50 maintained.

PML: percutaneous musculotendinous lengthening; GMFCS: gross motor function classification system; FIM: function independent scale; PRS: physician rating scale; OGS: observation gait analysis; PA: popliteal angle; MRI: magnetic resonance imaging; KEICd: knee extension at initial contact degree; GDI: gait deviation index; SFU: short follow-Up; GMFM: gross motor function measure; ROM: range of motion; FMS: function mobility scale.

**Table 4:** Complications of the included studies.

Study	Complications
Afaque et al.^19^	None
Ariyawatkul et al.^9^	N/A
Carbonell^10^	N/A
El Hage et al.^20^	Inadvertent partial section of adductor brevis in 12%
Erdal et al.^11^	N/A
Gordon et al.^14^	Recurvatum midstance: SFU:11%, LFU:17%
Hachache et al.^22^	Significant bleeding in 66% after PML (51% required hemostasis in open phase); common: minimal injury of adductor brevis; partial injury of obturator nerve (1 case)
Krupinski et al.^15^	None
Mansour et al.^21^	None
Mitsiokapa et al.^16^	None
Mozafari et al.^12^	Open: 1transitory peroneal nerve palsy, 1 stiff knee /recurvatum required distal transfer of rectus femoris. PML: 1 peroneal nerve palsy, 1 hamstring weakness /hip extension weakness /gluteus lurch—no recovery
Nazareth et al.^13^	PML: no complications. Open: (12%): temporary peroneal neuropraxia (1), hypersensitivity at the plantar aspect of the foot (3), heel pressure sore (3), wound infection (1).
Pierz et al.^17^	Recurvatum: 8 (3 had pre-surgery that improved but was still excessive, 1 had normal knee extension pre-surgery, and 4 had increased knee flexion pre-surgery).
Wild et al.^18^	Phase 2: 2.4% (hematoma (n=2), paresthesia (n=8), tight casts (n=4), transient flexion contractures (n=11), gracilis muscle rupture (n=1). Reoperation rate: 8%–13%.

N/A: not applicable; SFU: short follow-up; LFU: long follow-up; PML: percutaneous musculotendinous lengthening.

 PML indicated that increasing hip abduction was reported in three studies. One study included other muscles and obturator blocks; results were based on functional evaluations, and specific effects of PML on adductors were not provided.^16^

 Notably, two studies analyzed hip abduction after PML of adductor longus alone^20^ or with proximal gracilis,^22^ followed by open procedure (OP). Both showed improved abduction after PML with no additional benefit from the OP. Significant bleeding occurred in 66% of cases, with 51% requiring hemostasis via OP. Obturator nerve^22^ and adductor brevis injuries were also reported. Due to these complications, Hachache et al. concluded the procedure should be contraindicated.^22^

 In total, 7 of the 14 studies focused on hamstring PML.^9,12-14,17,19,21^ Procedures typically involved semitendinosus and gracilis, with semimembranosus or biceps added as needed. Notably, four studies assessed intraoperative PA; three studies included lateral hamstrings, one as OP, and two as PML. Evidently, one study performed PML on lateral hamstrings (biceps).^13^ An ultrasound-based study reported high peroneal nerve risk with lateral PML, recommending an open approach.^9^

 Notably, one study performed PML at multiple levels without isolating results for hamstrings.^16^ Specifically, three studies compared OP and PML,^12,13,19^ only one used 3D gait analysis data. These studies included patients from GMFCS level I to V. Follow-up ranged from 12 to 19.2 months. Procedures began with medial hamstrings, with lateral added if necessary. Concomitant procedures were reported in two studies: Mozafari et al. (Vulpius, Achilles lengthening, adductor tenotomy, posterior tibialis procedures)^12^ and Nazareth (femoral and tibial osteotomies, foot surgeries).^13^ All three studies showed improvement within each group, with no significant differences between techniques regarding PA, GMFCS, FIM, PRS, or OGS. Mozafari found both techniques equally effective in PA and GMFCS, with similar complication rates.^12^ Nazareth, using 3D gait analysis, found equivalent improvements in gait parameters, but greater anterior pelvic tilt in the PML group.^13^ Complications occurred only in the OP group, including temporary peroneal neuropraxia and plantar hypersensitivity.^13^

 One study analyzed medial hamstring PML followed by open exploration and fractional lengthening.^21^ The semimembranosus tendon was clinically identified pre-PML in only one--third of cases. Unintended fiber cuts over 50% occurred in 25% of semimembranosus cases and all semitendinosus cases (including six complete ruptures). PA gain was greater after the OP and unrelated to the extent of muscle division.^21^

 Notably, three studies used 3D gait analysis.^14,17,18^ Concomitant surgeries included gastrocnemius and bone procedures,^14^ Achilles lengthening and tibial osteotomy,^17^ and multilevel soft tissue and bone surgeries.^13^ Gordon et al.^14^ reported postoperative improvements in KEIC, stride length, velocity, PA, and GDI. Anterior pelvic tilt increased only in the short term. Recurvatum was observed in 11–17% of limbs. Pierz et al.^17^ found improvements in GMFCS I–III for PA, static knee extension, KEIC, and stance phase knee flexion, with pelvic tilt increase only in GMFCS III. Recurvatum occurred at 15%. Nazareth et al.^13^ found no significant kinematic differences between OP and PML groups, though both had decreased KFIC and greater knee extension in stance. 

 In total, three studies addressed this topic. Only one evaluated clinical outcome in about half of the original cohort, with a mean follow-up of 10.9 years, reporting 43% recurrence and no calcaneus deformity.^15^ Another study measured posterior superficial compartment pressure before and after Achilles tendon PML, showing a reduction of pressure after surgery.^10^ A third study assessed neurovascular safety and found sural nerve enlargement with degenerative changes in 16.7% and complete transection in 3.3% of cases on MRI.^11^

 Among the 14 studies included, 10 reported the GMFCS classification,^10,14-19,22^ and 7 of them assessed functional outcomes through different tools, such as the FMS, OGA, PRS, gait analysis, and GMFM.^13-19^ However, only a few provided detailed analysis or stratification of results according to GMFCS levels. 

 Afaque et al. analyzed patients classified from GMFCS I to V, comparing open and percutaneous hamstring lengthening. Both groups showed improvements in FIM, OGS, PRS, and PA, but there was no statistically significant difference between techniques, and only the PA results were stratified by GMFCS.^19^ Krupinski et al. evaluated PML of the Achilles tendon in GMFCS I and II through clinical examination, without stratifying results by GMFCS.^15^ Mitsiopaka et al. analyzed adductor and hamstring PML in patients from GMFCS I to IV, with 58% improving one GMFCS level and 8.6% two levels. GMFM scores improved in all patients, though results were not separated by GMFCS level.^16^ Nazareth et al. evaluated hamstring PML and open procedures in GMFCS I to IV using 3D gait analysis. Both groups demonstrated improved KFIC and increased maximum knee extension. However, several patients underwent concomitant procedures, including foot surgeries and femoral or tibial osteotomies, which likely influenced outcomes. No stratification by GMFCS level was provided.^13^ Pierz et al. was the only study that stratified outcomes by GMFCS level, using instrumented gait analysis to compare patients in levels I/II and III who underwent hamstring PML. Both groups showed improved KEIC and better mean knee flexion during stance. Stride length and walking velocity increased in patients with GMFCS I/II but not in those with GMFCS III. An increase in anterior pelvic tilt was noted postoperatively in GMFCS III patients. As in other studies, multiple simultaneous procedures were performed, which may have affected the results.^17^ Gordon et al. also employed instrumented gait analysis to evaluate PML of the hamstrings in patients with GMFCS I to III, comparing short-term (less than 18 months) and long-term (more than 18 months) follow-ups. Both groups showed improved KEIC, increased velocity and stride length, and an overall improvement in gait quality measured by the GDI. In the short-term group only, there was greater peak knee extension in stance, reduced plantar flexion at initial contact, and increased anterior pelvic tilt. Results were not stratified by GMFCS.^14^ Wild et al. assessed adductor, hamstring, and gastrocnemius PML in GMFCS I to IV, showing improvements in FMS (500 m) at final follow-up, but without separating results by GMFCS level.^18^ In summary, although GMFCS was frequently mentioned, few studies stratified or analyzed outcomes according to GMFCS levels. The overall heterogeneity of study designs, associated procedures, and methodological limitations substantially restricts meaningful comparison and interpretation of results. 

 In total, nine studies^11-19^ mentioned immobilizations in the postoperative period including long leg non-walking casts for 6 weeks^19^ and short leg casts for 3–6 weeks.^11,12,15,17,18^ Removable knee braces or immobilizers were used for 1–4 weeks.^13,14,16,18^ Ankle foot orthosis and night splints were used for a variable period of time (1–2 weeks—12 months).^12,13,16^

## DISCUSSION

 This systematic review identified 14 studies evaluating PML in children with CP. Although several studies were rated as high quality by NOS and presented a low risk of bias by RoB 2, the overall certainty of evidence remains low to very low according to GRADE criteria, due to methodological limitations, heterogeneity of outcome measurements, lack of randomized control groups, uncontrolled confounders (e.g., concomitant surgeries), and short follow-up periods, limiting conclusions on long-term outcomes like recurrence or over-lengthening. Few studies used standardized outcome tools or 3D gait analysis. 

 Half of the studies focused on hamstring PML, showing short- to mid-term improvements in joint ROM, particularly in PA. Some reported gains in GMFM, GMFCS, FMS, and gait parameters (e.g., KEIC, GDI, velocity),^12,14,16,18^ with mild increases in pelvic tilt and recurvatum (up to 17%).^14^ Most studies were retrospective, lacked control groups, included additional procedures, and had limited follow-up.^12,13,17^

 Notably, three studies compared PML of hamstrings with OP and generally showed no significant differences in functional or kinematic outcomes.^12,13,19^ Some advantages of PML (e.g., reduced bleeding, shorter surgery time, and quicker recovery) were suggested, though only Mozafari et al.^12^ provided specific data. Nazareth et al.^13^ used 3D gait analysis and reported no significant differences in knee kinematics but found anterior pelvic tilt >10° in 23% of PML cases vs. 14% of OP cases. However, limitations such as retrospective design, unequal groups, and concomitant surgeries restrict interpretation. 

 Although the Achilles tendon is probably the most common PML performed, we were able to find only one study reporting specific results. Krupinski et al.^15^ analyzed just 50% of their initial sample after 10.9 years. Using only clinical observation of the gait, the authors reported high recurrence of equinus deformity despite good initial correction, no overcorrection, and complete patient satisfaction. This is a very important matter to be considered, since long-term studies suggest serious outcomes after Achilles lengthening. Dietz et al.^23^ found crouch and need for anterior floor-reaction orthoses in 41% of diplegic and 50% of quadriplegic patients, often diagnosed up to 10 years after surgery. A Delphi consensus concluded Achilles (Zone 3) lengthening is contraindicated in diplegia.^24^ Muscle-tendon architecture changes may underlie these issues. Wren et al. showed that children with CP have longer tendons and shorter muscle bellies than peers; surgery increases tendon length but doesn’t restore architecture, possibly leading to plantarflexor weakness and deformity.^25^ Besides this study on PML of Achilles, we found Wild et al.^18^ reporting ankle dorsiflexion improved in patients with true equinus but not consistently in crouch gait, suggesting limited benefit when equinus is compensatory. In this study, however, PMTL was also performed in hamstrings and adductors, limiting the analysis of specific results. 

 Although PML is considered minimally invasive and cosmetically favorable, it carries risks: uncontrolled lengthening, nerve injury, and unplanned muscle/tendon damage.^9,11,18,21,22^ Anatomical and imaging studies raised concerns about nerve injury: sural, obturator, and peroneal nerves were affected in some cases.^9,11,22^ Peroneal nerve post lateral hamstring PML was shown to be related to direct contact (15.6% of the cases)^9^ or postoperative knee hyperextension.^26^ Sural nerve enlargement and even complete transection were seen on MRI in 16.7% and 3.3% of ankles, respectively.^11^ Inadvertent rupture of the semitendinosus and semimembranosus was also reported.^21^

 The choice between PML and OP is not well defined in the reviewed literature and often depends on individual clinical judgment. PML may offer benefits such as being minimally invasive, quicker, less painful, and more cosmetically favorable. However, it carries risks such as limited control over-lengthening and the inability to visualize nearby neurovascular structures. Surgical positioning and the broader operative plan also influence technique selection. Ultimately, appropriate patient selection and surgical goals are key to determining the optimal approach. 

 Despite promising results, evidence supporting PML in CP is limited. Most studies were retrospective, small, with short follow-up, limited functional assessment, and methodological heterogeneity. These constraints mirror broader challenges in pediatric surgical research. 

 Given the risks, especially the risk of over-lengthening and functional deterioration, stronger evidence is needed before broadly recommending PML. Future studies should prioritize RCTs with adequate sample size, long-term follow-up, standardized classifications (e.g., GMFCS), and objective outcome tools such as 3D gait analysis. Investigating ideal timing, patient selection, long-term participation, and quality-of-life outcomes is essential. 

 This review may be subject to reporting bias, as negative or inconclusive studies may be underrepresented. Most included studies were published in English, introducing potential language bias. Positive findings in small studies may be preferentially published, contributing to publication bias. These factors could overestimate the benefits or underestimate the risks of PML in children with CP. No funnel plot analysis was conducted due to the limited number of RCTs (n=3), but this limitation was acknowledged. Selective outcome reporting could not be formally assessed due to the lack of pre-registered protocols for most included studies. Furthermore, the heterogeneity of study populations, with few studies stratifying participants according to GMFCS levels, limits the ability to evaluate functional gains versus losses across different functional severity groups. Future research with longer follow-up periods, more homogeneous samples, and standardized functional outcome measures is warranted to better inform therapeutic guidance. These issues warrant caution when interpreting the results. 

 In conclusion, despite methodological limitations, available observational evidence consistently suggests that PML can provide meaningful short- to mid-term improvements in children with CP. However, due to the lack of long-term results, limited adjustment for confounding factors, inconsistent use of objective gait analysis across studies, and heterogeneity of study populations with few stratified by GMFCS levels, the evidence is not sufficient to fully recommend the procedure. Particular caution is warranted for Achilles tendon lengthening, where over-lengthening can cause severe functional problems. Future research with well-designed prospective trials should prioritize standardized patient selection, homogeneous samples, stratification according to functional severity (GMFCS levels), standardized outcome measures, longer follow-up, and careful assessment of functional gains versus losses, as well as clearer analysis of concurrent procedures, to strengthen the evidence supporting surgical decision-making in this population. 

## Data Availability

The database that originated the article is available, with the corresponding author.
